# Involvement of Arabidopsis BIG protein in cell death mediated by *Myo*-inositol homeostasis

**DOI:** 10.1038/s41598-020-68235-4

**Published:** 2020-07-09

**Authors:** Quentin Bruggeman, Florence Piron-Prunier, Frédérique Tellier, Jean-Denis Faure, David Latrasse, Deborah Manza-Mianza, Christelle Mazubert, Sylvie Citerne, Stéphanie Boutet-Mercey, Raphael Lugan, Catherine Bergounioux, Cécile Raynaud, Moussa Benhamed, Marianne Delarue

**Affiliations:** 1 Université Paris-Saclay, CNRS, INRAE, Univ Evry, Paris Diderot, Sorbonne Paris-Cité, Institute of Plant Sciences Paris-Saclay (IPS2), 91405 Orsay, France; 2 Institut Jean-Pierre Bourgin, INRAE, AgroParisTech, Université Paris-Saclay, 78000 Versailles, France; 3 Institut de Biologie Moléculaire Des Plantes, Unité Propre de Recherche 2357 CNRS, Université de Strasbourg, 12 rue du Général Zimmer, 67084 Strasbourg Cedex, France

**Keywords:** Plant development, Plant physiology, Plant stress responses

## Abstract

Programmed cell death (PCD) is essential for several aspects of plant life. We previously identified the *mips1* mutant of *Arabidopsis thaliana*, which is deficient for the enzyme catalysing myo-inositol synthesis, and that displays light-dependent formation of lesions on leaves due to Salicylic Acid (SA) over-accumulation. Rationale of this work was to identify novel regulators of plant PCD using a genetic approach. A screen for secondary mutations that abolish the *mips1* PCD phenotype identified a mutation in the *BIG* gene, encoding a factor of unknown molecular function that was previously shown to play pleiotropic roles in plant development and defence. Physiological analyses showed that BIG is required for lesion formation in *mips1* via SA-dependant signalling. *big* mutations partly rescued transcriptomic and metabolomics perturbations as stress-related phytohormones homeostasis. In addition, since loss of function of the ceramide synthase LOH2 was not able to abolish cell death induction in *mips1*, we show that PCD induction is not fully dependent of sphingolipid accumulation as previously suggested. Our results provide further insights into the role of the BIG protein in the control of MIPS1-dependent cell death and also into the impact of sphingolipid homeostasis in this pathway.

## Introduction

*Myo*-inositol (MI) is a ubiquitous molecule and the precursor for biosynthesis of many inositol-derivatives such as inositol phosphates, phosphatidylinositol (PtdIns) phosphate, or specific classes of sphingolipids that play critical and diverse roles in vesicle trafficking, hormone signalling, and biotic and abiotic stress responses^1, 2^. The rate-limiting step of MI synthesis is catalysed by l-*myo*-inositol 1-phosphate synthase (MIPS), using Glucose-6-P as a substrate. This reaction is followed by dephosphorylation of l-*myo*-inositol 1-phosphate into MI. These two reactions together, called the Loewus pathway, are the only known route for MI biosynthesis^3^.

Three genes encoding MIPS isoforms have been identified in the Arabidopsis genome^4, 5^, among them MIPS1 is responsible for most of MI biosynthesis in leaves. Pleiotropic developmental defects such as reduced root growth or altered venation in cotyledons have been described for the *mips1* loss of function mutant^6–8^. One of its most striking features is the light intensity-dependent formation of leaf lesions due to Salicylic acid (SA)-dependent Programmed Cell Death (PCD), revealing the importance of MI or inositol derivatives in the regulation of these processes. However, how light affects *MIPS* transcript level and inositol biosynthesis was an open question for a long time until the demonstration that the two light signalling proteins, FAR-RED ELONGATED HYPOCOTYL 3 (FHY3) and its homolog, FAR-RED IMPAIRED RESPONSE 1 (FAR1), were able to directly bind *MIPS1* promoter and to activate its expression, thereby promoting inositol biosynthesis to prevent light-induced SA-dependent cell death^9^.

Because MI is used to synthetize numerous compounds in the cell, including PtdIns, a link between *mips1* PCD and an alteration of sphingolipid homeostasis has been hypothesized by^7^. Sphingolipids are a class of lipids present ubiquitously both in eukaryotes and bacteria structurally characterized by a sphingoid base acyl chain amide linked to a fatty acid (FA), to form the basic unit of all sphingolipids: the ceramide (Cer). Complex sphingolipids are formed by a ceramide or hydroxyl ceramide with the addition of a polar head such as a glycosyl group or a combination of glycosyl and inositol-glucuronic acid groups to form respectively Glucosylceramide (GlCer) and Glycosylinositol phosphoceramide (GIPC). Variability in the length of the sphingoid LCB and the FA, in the number of saturations, in the degree of hydroxylation, and the diversity of polar head groups are responsible of the high structural diversity displayed by sphingolipids^10^. Hence, plant sphingolipids can be divided into four main classes: ceramides, GlCers, GIPCs, and free LCBs^10^. Several reports describe the importance of sphingolipids in biotic stress response during which they induce PCD and SA signalling (reviewed by^11^). Recent work addressing Arabidopsis ceramide synthase specificity toward FA chain length established that LAG ONE HOMOLOG (LOH) 1/3 preferentially use VLCFA as substrates whereas LOH2 rather forms C16 containing ceramides^12^. *LOH2* overexpression induced as expected a strong enrichment in ceramide molecular species with 16:0 FA but also additional phenotypes reminiscent of that of *mips1*, including increased SA concentrations, PR1 gene expression and localized PCD^13^. Likewise, it has been recently shown that an increase of sphingolipids content in ceramidase loss-of-function mutants in Arabidopsis, is correlated with spontaneous cell death accompanied by higher level of SA and other related-stress phytohormones^14^. Consistently, it has been observed that the cell death phenotype of *mips1* mutant correlated with elevated levels of ceramides and hydroxyceramides, likely because the conversion of ceramides in inositol phosphoryl ceramide is compromised in this mutant due to the inhibition of MI production^7^.

In order to gain insight into the molecular mechanism involved in MI-dependent cell death, a screen for extragenic secondary mutations that abolish lesion formation in *mips1-1* using ethyl methanesulfonate (EMS) mutagenesis has been previously performed. Several *suppressors of mips1* (*somi*) mutants were thus identified such as *somi1*, mutated in the Hexokinase 1 (HXK1) enzyme and that was able to restore MI accumulation in the *mips1* mutant^15^, or *somi3*^16^, that rescues lesion formation through reduction of chloroplast activity. Following a candidate gene approach, screen for mutants that display transcriptomic profiles opposing that of the *mips1* mutant, has led to the identification of the Cleavage and Polyadenylation Specificity Factor 30 (CPSF30) as a key component of plant PCD required for the proper expression of a large set of genes involved cell death, defence and abiotic stress responses. We also showed that mutation of *CPSF30* suppresses cell death in other lesion-mimic mutants (LMM), including the *mitogen-activated protein kinase 4* (*mpk4*) mutant^17^, revealing that CPSF30 is a more general regulator of the SA pathway rather than a specific regulator of MI-mediated PCD.

Here, we report another mutation, in a gene named *BIG*, able to suppress *mips1* light-dependent PCD. We demonstrated an epistatic relationship between *mips1* and *big* mutations and that PCD triggered by a decrease in MI content can be reverted by the *big* mutation, possibly through the suppression of SA production.

This gene, *BIG*, encoding a calossin-like protein, is one of the longest genes in the Arabidopsis genome and a large number of *BIG* alleles have been identified in independent genetic screens. One mutant in the *BIG* gene, *tir3-1*, was originally isolated for its resistance to auxin transport inhibitor NPA^18^. Other *big* mutants were subsequently identified, exhibiting a variety of morphological defects and resistance to the auxin’s effect on endocytosis^19,20^; reduced response to cytokinin, ethylene, and gibberellins^21^; corymb-like inflorescences^22,23^; or characterized by mis-regulation of mitochondrial stress signalling^24^. BIG has been also described for its involvement in the circadian clock control^25^ and in elevated CO_2_-induced stomatal closure^26^. Recently^27^, demonstrated that BIG represents a new regulator of the Jasmonic Acid (JA) pathway and a point of convergence for the interactions of JA and others hormones. BIG deficiency promotes JA-dependent gene induction, increased JA production but restricts the accumulation of SA.

Here, we show, by genetic approaches, an epistatic relationship between *mips1* and *big* mutants and that PCD triggered by a decrease in MI content and SA accumulation can be reverted by a *big* mutation. In addition, loss of function of the ceramide synthase LOH2 was not able to abolish cell death induction in *mips1*, showing that PCD induction is not fully dependent of sphingolipid accumulation as previously suggested.

## Results

### *somi2* suppresses cell death and partially restores a wild-type phenotype in the *mips1* mutant

One remarkable aspect of the *mips1* mutant phenotype is the spontaneous lesion formation observed when plants are growth in long-day (LD) conditions, whereas when grown under short-day (SD) conditions, mature *mips1* mutants are indistinguishable from the wild type^6–8^. A screen for suppressors of *mips1-1* using EMS mutagenesis has been previously performed in order to better understand how MIPS1 negatively regulates cell death^15^. Among the 50 *somi* mutants identified, the *somi2* mutant was further characterized in detail in this study.

**Figure 1:**
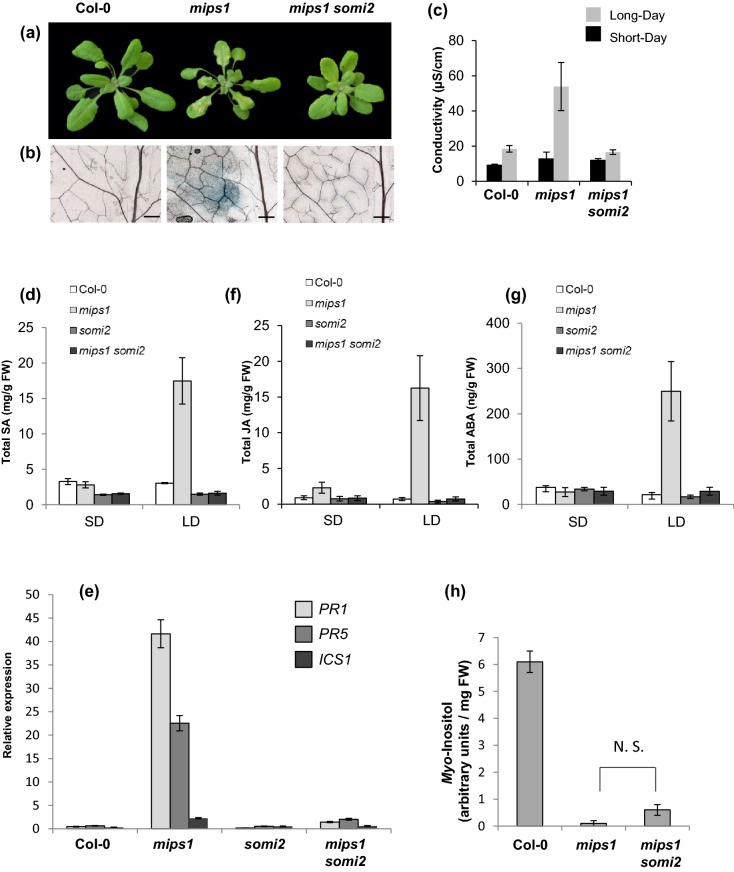
The *somi2*-mediated suppression of SA-dependent cell death in *mips1* Arabidopsis mutant. (**a**) Suppression of *mips1*-mediated cell death in the *mips1 somi2* background. Plants were grown 1 week in vitro and then 14 d in SD conditions in soil and photographed 5 d after transfer to LD conditions. Bar = 1 cm. (**b**) Trypan blue staining of wild-type, *mips1*, *somi2*, and *mips1 somi2* leaves. Bars = 2 mm. (**c**) Ion leakage assays, with means and SD calculated from six discs per treatment with three replicates within an experiment in LD and SD conditions (**d**, **f** and **g**) respectively, total SA, JA and ABA levels in the indicated genotypes, with means and SD calculated from four biological replicates grown for 14 days in SD conditions and transferred to LD conditions for 4 days (LD) or stayed in SD (SD). (**e)** Real-time RT-PCR analysis of *PR1* (*AT2G14610*), *PR5* (*AT1G75040*) and *ICS1* (*AT1G74710*) expression in rosette leaves of plants with the indicated genotypes, 4 days after transfer to LD conditions. Transcript abundance is expressed relative to *UBQ10* (*AT4G05320*) transcript abundance. Average relative quantities 6 SD (n = 3). (**h**) GC–MS analysis of myo-inositol contents in rosette leaves of the indicated lines. Samples were harvested 4 days after transfer in LD conditions. Standard deviations were calculated from four biological replicates. There was Not Significant (N.S.) different values according to Student’s test (P > 0.02). FW, Fresh Weight.

Four days after transfer in restrictive LD conditions, cell death was observable on *mips1-1* leaves, whereas the *mips1-1 somi2* double mutant did not show any cell death (Fig. 1a). Staining with Trypan blue (Fig. 1b) and measurements of ion leakage (Fig. 1c) confirmed this observation.

Since lesion formation in *mips1-1* is SA dependent, we measured the effect of the *somi2* mutation on SA content in the *mips1-1* background. As previously reported^6^, free and total SA contents were similar in the different *mips1-1* and wild-type plants under permissive conditions (i.e. SD), but dramatically increased in the *mips1-1* mutant 4 d after transfer to restrictive conditions (Fig. 1d). This increase in SA was abolished in *mips1-1 somi2* double mutants. We also quantified scopoletin, a phytoalexin that confers enhanced resistance against pathogens, establishing a hypersensitive cell death in response to bacterial pathogens^28^. Similarly to SA, the increase in scopoletin levels observed in *mips1* was suppressed in the *mips1-1 somi2* background (Fig. S1). In addition, the *somi2* mutation led to a dramatic down-regulation of the SA pathogenesis-related marker transcripts *PATHOGENESIS-RELATED1* (*PR1*), *PR5* and *ISOCHORISMATE SYNTHASE1* (*ICS1*) (Fig. 1e) in *mips1-1 somi2* compared to *mips1-1*.

To test if changes in homeostasis of other phytohormones, related to stress or developmental defects, can be related to *mips1* phenotype, and its suppression, we also analysed JA, abscisic acid (ABA) and auxin content in these mutants. We found that the content of both stress-related hormones (i.e. JA and ABA) were largely increased in the *mips1-1* mutant 4 d after transfer to restrictive conditions, whereas these contents were similar to will-type levels in *somi2* and *mips1 somi2* background (Fig. 1f, g). In addition, no modification in the auxin content was observed in any of the mutants (Fig. S2).

Defects in primary root development and alteration in cotyledon morphology constitute the two others striking phenotypes of the *mips1* mutant^6–8,29^. The short primary root phenotype displayed by *mips1-1* was not suppressed in the *mips1-1 somi2* mutant (Fig. S3). To examine whether the defects in cotyledons morphology was suppressed by the *somi2* mutation, we categorized cotyledon abnormalities into 4 classes according to the severity of the phenotype (Fig. S4). The *mips1-1 somi2* mutant displayed almost similar proportion of plants with severe cotyledon defects as the *mips1-1* mutant. All these results suggest that SOMI2 factor is specifically involved in the cell death-related defect of the *mips1* mutant and not in other cellular processes requiring MI.

### Cell death suppression in *mips1* is not due to a restoration of MI content

In the *mips1* mutant, the induction of PCD was attributed to the reduced MI or galactinol accumulation, since treatment of mutants with either compounds was able to suppress lesion formation^6^. Hence, using gas chromatography-mass spectrometry (GC–MS) analysis, MI content was quantified in the different genetic backgrounds. While as expected, MI content was strongly decreased in the *mips1-1* mutant compared to the wild type, accumulation of this compound was also severely impaired in *mips1 somi2* double mutant (Fig. 1h). Measurements of raffinose led to the same results: the drastic reduction of this molecule in *mips1* was not restored to a wild-type level by the *somi2* mutation (data not shown).

### *SOMI2* corresponds to the *BIG* gene

**Figure 2:**
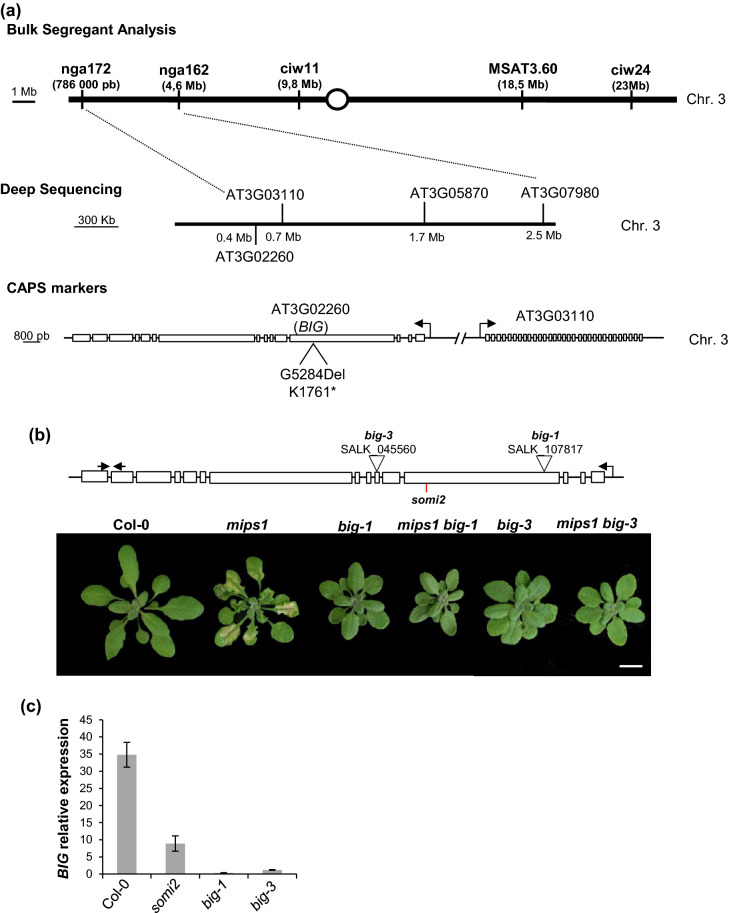
Identification of the *somi2* mutation. (**a**) Bulk segregant analysis was performed using F2 from the cross between *mips1-1 somi2*, in the Col-0 background, and *mips1-2*, in the Ws background, and revealed that *somi2* was flanked by markers nga172 and nga162 on chromosome 3 (Chr. 3). High-throughput sequencing of nuclear DNA obtained from a bulk of F2 plants without lesions in LD identified four candidate genes (*AT3G02260*, *AT3G03110*, *AT3G05870* and *AT3G07980*). CAPS markers were used to remove SNP mutations not linked to the *somi2* locus and 1 gene remained after this step: *AT3G02260*, encoding BIG. Mutations introduced an early stop or an amino acid change of alanine to valine, in BIG. (**b**) Suppression of *mips1*-mediated cell death in *mips1 big-1 and mips1 big-3* backgrounds. Plants were grown 1 week in vitro and then 14 d in SD conditions in soil and photographed 5 d after transfer to LD conditions. Bar = 1 cm. (c) RT-qPCR analysis of the *BIG* expression in indicated lines in LD conditions. Transcript abundance is expressed relative to UBQ10 (AT4G05320) transcripts abundance.

To map the *somi2* mutation, an allelic mutant of *mips1-1*, the *mips1-2* allele, in the Wassilewskija (Ws) background, was crossed with *mips1-1 somi2* plants in the Columbia-0 (Col-0) background. A bulk segregant analysis (BSA) was performed using F2 plants without (n = 11) or with lesions (n = 11) in LD conditions and revealed that *somi2* was flanked by microsatellite markers nga172 and nga162, on a region of ≈ 4 Mb on chromosome 3 (Fig. 2a). Further mapping was performed by high-throughput sequencing of nuclear DNA obtained from a bulk of 116 F2 plants without lesion in LD. Sequence analysis using the Col-0 genome as reference revealed that in the region previously mapped by BSA, four candidate genes (AT3G02260, AT3G03110, AT3G05870 and AT3G07980) presented a mutation in 100% of paired-end reads. After two backcrosses of *mips1-2 somi2* with the *mips1-2* single mutant to clean background mutations, we used cleaved amplified polymorphic sequence (CAPS) markers and crosses with T-DNA insertion lines for candidate genes to identify the causal mutation. Through this approach, a deletion of a guanine leading to the apparition of a stop codon was detected in the 4th exon in *AT3G02260*, a large gene spanning 17,506 bp named *BIG*, encoding a protein with unknown biochemical function (Fig. 2a). *mips1-1 big-1* double mutants did not show any lesion (Fig. 2b), suggesting that a loss of function of BIG was able to suppress *mips1*-dependent cell death phenotype. The same result was obtained with crosses with another knock-out allele of *big* mutant, *big-3* (Fig. 2b). To confirm this result, complementation tests were performed between the *somi2* mutation and the *big-1* and *big-3* mutations. First, in order to obtain *somi2* quasi-isogenic lines, the *mips1 somi2* double mutant was crossed during 3 successive generations with wild-type Col-0 plants. Crossing of *somi2* with either *big-1* or *big-3* homozygous mutants resulted in the same compact rosette phenotype characteristic of *big* mutants without lesion in the first filial generation (Fig. S5), confirming that *somi2*, *big-1* and *big-3* were allelic mutations within the same gene.

Quantitative RT-PCR analysis of *BIG* expression in these different genetic backgrounds showed that this gene was strongly under-expressed in *big-1* and *big-3* mutants and also in *somi2* mutant (Fig. 2c). As the *somi2* mutation results in a single nucleotide deletion in an exon, this therefore suggests that the BIG protein may be directly or indirectly involved in controlling its own transcription or the stability of its messengers. Another hypothesis would be that the early stop codon induced by the *somi2* mutation is detected by the N on-sense Mediated Decay (NMD) mechanism, which is involved in the degradation of mRNAs with a premature stop codon in eukaryotes^30^.

### Disruption of BIG partially rescues the transcriptional reprogramming of the *mips1* mutants

To further explore the suppression of the *mips1* phenotype by the *somi2* mutation, we performed messenger RNA sequencing (RNA-seq) using rosette leaves of *mips1 somi2* double mutant grown in restrictive LD conditions together with wild-type, *mips1* and *somi2* single mutants. Principal component analysis (PCA) revealed a clear clustering of samples by genotype (Fig. S6). We next determined the Differentially Expressed Gene (DEG) set in each mutant compared to wild type, and performed a gene ontology (GO) term analysis focused on biological processes.

**Figure 3:**
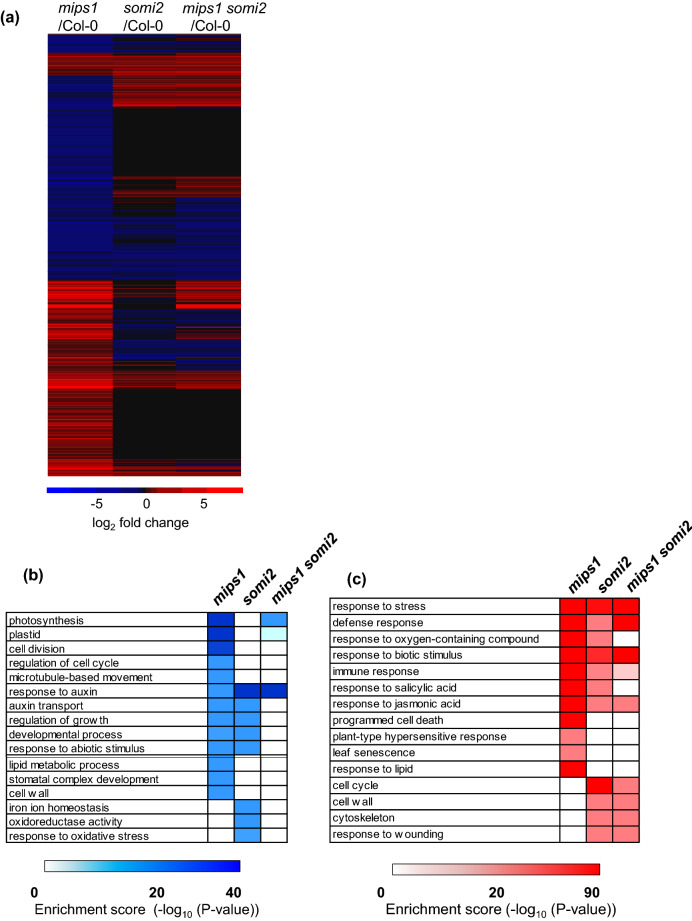

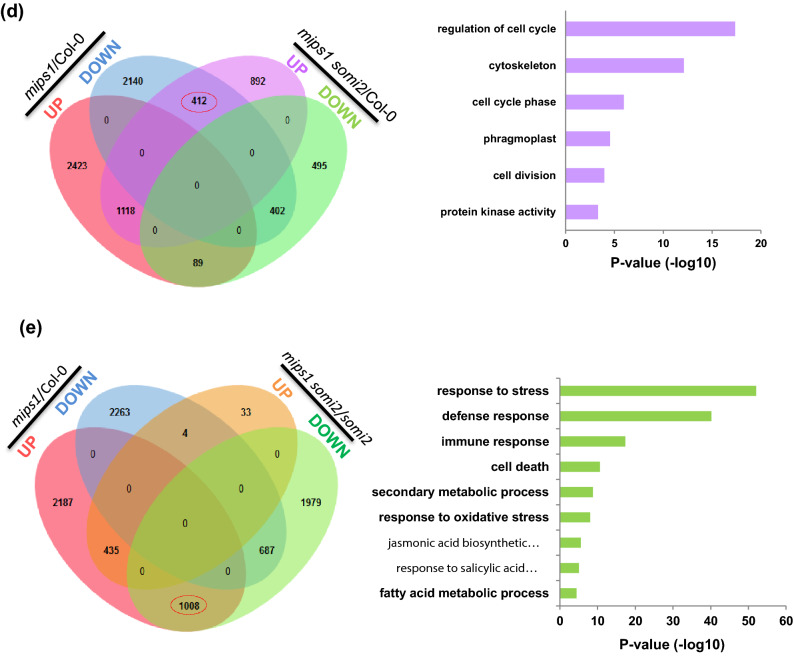
Characterization of gene networks deregulated by the *mips1* and *somi2* mutations. (**a**) Hierarchical clustering of genes differentially expressed between each mutants and the WT Col-0. Genes for which at least one comparison showed differential expressions were retained (p value < 0.05). Red and blue correspond to up- and down-regulated expression, respectively. The heat map was realized using heatmap.2 package of the R software (**b**, **c**) Over-represented Genes Ontologies analysis of under-expressed (**b**) and over-expressed (**c**) genes in each genotype. Analyses were performed with the agriGO online software^31^. The colour represents value of − log P. (**d**, **e**) Venn diagrams showing the differentially regulated genes in common between the different comparisons. The *mips1* mutant was compared to the Col-0 reference and the *mips1 somi2* double mutant was compared to the Col-0 reference (**d**) or the *somi2* mutant (**e**). Selected enriched biological processes (p value < 0.05) of the set of genes circled in red, is indicated on the right of the diagram.

As observed previously^6^, mutation in the *MIPS1* gene resulted in large changes of the transcriptome profile with 3,630 and 2,954 transcripts respectively up-regulated and down-regulated compared to the wild-type Col-0. These variations were much higher than those previously described^6^, likely due to the improvement of the transcriptomic analysis method used (RNA-seq technology compared to MicroArray data). We also found a significant number of DEGs in *somi2* single mutant (1628 up-regulated and 505 down-regulated) and *mips1 somi2* double mutant (2,422 up-regulated and 986 down-regulated) compared with wild-type Col-0 with a number of DEG in the *mips1* mutant displaying a lower fold change in *somi2* and *mips1 somi2* compared to the WT (Fig. 3a).

To identify the types of gene networks deregulated by the combination of the *mips1* and *somi2* mutations, the data sets were mined with the software tools AgriGO^31^. In the *mips1* mutant, overrepresented Gene Ontology (GO) terms among under—expressed genes are significantly enriched in photosynthesis and the organization of plastids and light-harvesting complex, results consistent with previous *mips1* transcriptomic and genetic analyses showing that the chloroplastic function was severely impaired in *mips1* mutants under restrictive conditions (Fig. 3b)^6,16^. In addition, response to hormone, and specifically auxin response and transport, and also lipid metabolism, cell wall, microtubules and cell cycle GO terms were also enriched among this class of down-regulated genes. Transcriptomic analysis of the *somi2* mutant indicated that only 23% of DEG were down—regulated including among them the *BIG* gene, which confirms our previous results of quantitative RT-PCR analysis. GO analysis of the genes showed a significant enrichment in transcripts related to growth, development and auxin signalling pathways (Fig. 3b) which is consistent with the reported function of BIG in auxin signalling response^32^. Likewise, as described previously by^27^, ethylene biosynthesis genes (i.e. *ACS5*, *ACS8*, *ACO3*) displayed much lower expression levels in *somi2* mutants than in the WT. A set of genes involved in iron homeostasis, oxidoreductase and response to oxidative stress were also down-regulated in the *somi2* background. It is worth noting that genes involved in photosynthesis, ethylene biosynthesis and auxin transport are still down-regulated in *mips1 somi2* double mutant, indicating that these pathways are not fully restored in this background (Fig. 3b). Moreover, a set of genes involved in plant sphingolipid metabolism, according to the classification established by^33^, were specifically down-regulated in *mips1* background. Remarkably, all these genes, (listed in Table S1), encode activities for the elongase complex involved in VLCFA synthesis as 3-*KETO ACYL-COA SYNTHASE* (*KCS*) genes, *PASTICCINO2* (*PAS2*), *ECERIFERUM 10* (*CER10*) and also two *FATTY ACID HYDROXYLASE* (*FAH*), involved in the synthesis of hydroxyl ceramides.

Correlated to the phenotype of *mips1* mutant, genes involved in stress, defence, immune response, programmed cell death, senescence, response to biotic stimulus, SA and oxidative compounds were largely over-represented among genes over-expressed in *mips1* background also confirming our previous results (Fig. 3c)^6^. Among these set of genes, it can be noted that the *LOH2* ceramide synthase gene is specifically over-expressed in *mips1* mutant as well as *PR1* and *PR5*, confirming the results described above. Some similar GO terms (defence, response to stress and biotic stimulus) were also found for genes overexpressed in *somi2* and also in *mips1 somi2* double mutant (Fig. 3c) and thus are neither specific to MIPS1 activity nor to lesion formation, although the addition of the *somi2* mutation in *mips1* background tends to reduce the number of defence-related genes among over-represented genes.

In the *somi2* background, 76% of the DEGs displayed an increased expression compared to the wild type. As expected from previous characterization of *big* alleles^27^, genes involved in JA biosynthesis and signalling pathway, as respectively *LOX2*, *JAZ1*, *JAZ5, JAZ7 and JAZ10* or in ethylene response were up-regulated. More generally, GO analysis of these genes showed a significant enrichment in response to stimulus, immune and defence response, anthocyanin production, response to ethylene, wounding and JA stimulus (Fig. 3c). Additional over-represented terms, not found in the *mips1* background, corresponded to cell cycle, cytoskeleton and cell wall, GOs that can be correlated with the developmental defects observed in the leaves of the *somi2* mutant and that are still persistent in *mips1 somi2* (Fig. 2b).

In order to check whether the expression of a significant number of genes were inversely controlled according to the presence or absence of the functional BIG peptide, a cross comparison between up and down-regulated genes was performed. Overall, the heat map shows that the transcriptomic profiles of the diverse lines, compared to the wild-type Col-0, can be grouped into two main categories: *mips1* in one group and *somi2* and *mips1 somi2* in the other group. Respectively, 35% and 32% of up- and down-regulated genes in *mips1* background displayed a wild type expression level in *somi2* and *mips1 somi2* mutants (Fig. 3a). This clustering correlated with the presence of HR-like phenotypes and also confirmed an epistatic relationship between *mips1* and *somi2*, with *somi2* being epistatic over *mips1* and it can suggest that the loss of BIG activity is able to dampen the impact of the *mips1* mutation on the Arabidopsis transcriptome.

Whereas the vast majority of over-expressed genes in *mips1* background does not display down-regulation in *mips1 somi2* background compared to the wild type, a set of 412 genes down-regulated in *mips1* background were over-expressed in *mips1 somi2* compared to the wild-type (Fig. 3d). GO terms of this list revealed a clear enrichment in genes involved in cell cycle functions (Table S2), and more precisely in the control of cell cycle arrest, like Cyclin genes, indicating that lesions formations is correlated with a cell cycle arrest whereas its suppression corresponded to a transcriptional switch of the cell cycle machinery.

In order to confirm that the *mips1* mutation requires BIG to induce cell death and defence responses, we focused on genes that displayed altered expression in *mips1* compared to wild type, and whose expression was modified in *mips1 somi2* compared to *somi2* (Fig. 3e). In total, 27% of genes upregulated in *mips1* compared to wild type, were downregulated in *somi2* background and therefore associated with the phenotype of the suppression of cell death. As expected, GO term analysis revealed that within this set of gene, genes associated with defence, immune and cell death response were largely over-represented (Fig. 3e). However, only 4 of the 2,954 down-regulated genes in *mips1* mutant compared to the wild-type were up-regulated when compared within the *somi2* background (Fig. 3e) indicating that the cell death phenotype due to the absence of MIPS1 protein is probably mainly associated with a transcriptional switch leading to an overexpression defence and immune-response related genes.

### The *somi2* mutation suppresses sphingolipid accumulation in *mips1* mutant

The relative accumulation of ceramides and their derivatives appear as key elements for the control of plant PCD^10,33^. This was further supported by the detection of elevated levels of ceramides and hydroxyceramides in the *mips1* mutant (Donahue et al. 2010). In order to check whether the sphingolipid content is affected by the *somi2* mutation in the *mips1* background, quantification of ceramide, hydroxyceramide, GlCer and GIPC contents was performed in single and double mutant lines.

**Figure 4:**
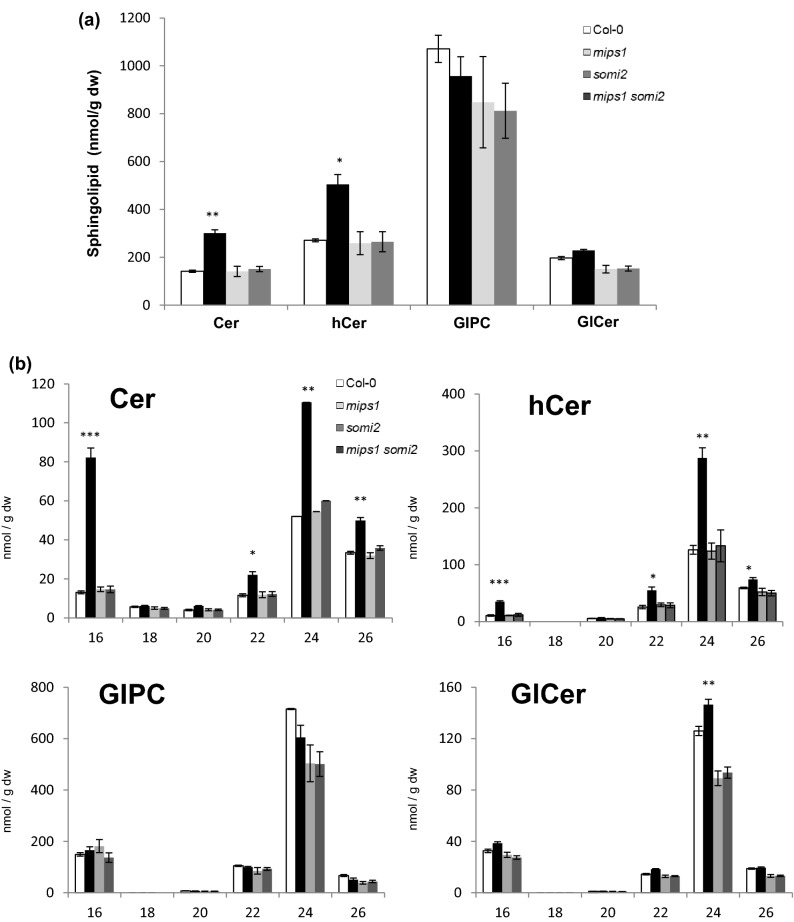
Sphingolipid contents alterations in *mips1*, *somi2* and *mips1 somi2* double mutants compared to the wild-type Col-0. (**a**) Comparison of total amount of ceramide (Cer), hydroxyceramides (hCer), glucosylceramides (GlCer) and glycosyl inositol phosphoryl ceramide (GIPC). dw, Dry Weight. (**b**) Sphingolipid contents detailed according to the four different classes (Cer, hCer, GlPC and GlCer) and the length of the fatty acid chain. Arrows indicate major increases. The means values of three biological replicates are shown ± SD. Asterisks indicate significant differences between WT control plants and indicated genotypes according to Student’s *t-*test (*P ≤ 0.05; **P ≤ 0.01; ***P ≤ 0.001).

In plants, the amounts of sphingolipid classes tend to vary in a species and tissue-dependent manner. In agreement with previous studies^34^, our sphingolipid profile quantification confirmed that the GIPCs are the predominant class in Arabidopsis leaves. In addition, these analyses indicated an approximately twofold increase in ceramide and hydroxyceramide contents in the *mips1* mutant compared to the wild-type (Col-0) without significant changes in levels of GlCer and GIPC (Fig. 4a). These increases were abolished in the *mips1 somi2* double mutant, whereas *somi2* mutants displayed similar sphingolipids levels as the wild-type (Fig. 4a).

To gain further insight into the composition of different classes of molecules in terms of length of the fatty acid chain, specific quantifications of molecular species were performed (Fig. 4b). This revealed that the elevated amounts measured in *mips1* mutant, were mainly due to an increase of C16, C22, C24 and C26 fatty acid ceramides and hydroxyceramides. It confirmed also that these amounts did not change in *somi2* and *mips1 somi2* mutants compared to the wild-type Col-0. These results correlated sphingolipids accumulation and the appearance of HR-like phenotypes in the *mips1* mutant and also confirmed an epistatic relationship between *mips1* and *somi2*, with *somi2* being epistatic over *mips1*.

Since C16-sphingolipid were specifically associated with PCD and because RNA seq data analysis of this work revealed that the *LOH2* ceramide synthase gene was specifically up-regulated in *mips1* background, without significant change in *somi2* and *mips1 somi2* mutants compared to the wild-type, we checked whether a loss of function of the *LOH2* ceramide synthase gene was able to abolish *mips1*-mediated cell death by interfering with C16 containing ceramides synthesis.

**Figure 5:**
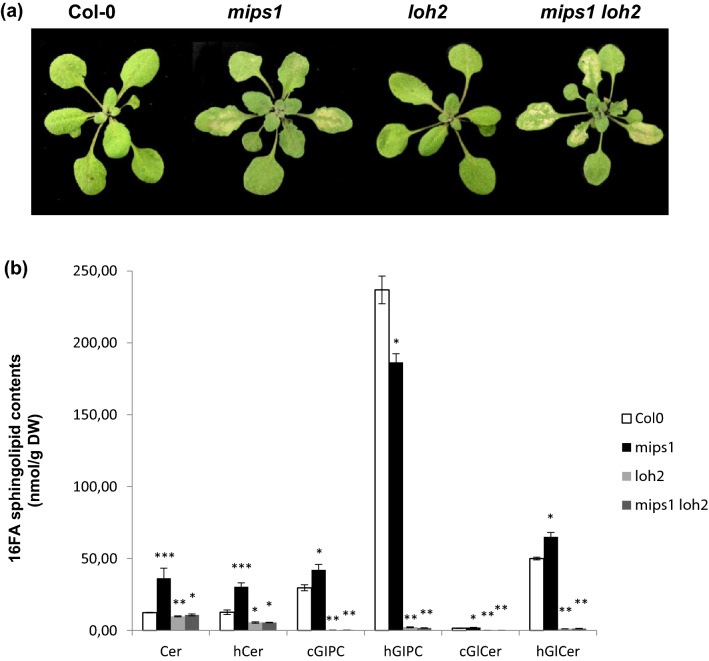
Phenotypical and sphingolipid content alterations in *mips1*, *loh2* and *mips1 loh2* double mutants compared to the wild-type Col-0. **(a**) *loh2* mutation is not able to suppress *mips1*-mediated cell death. Plants were grown 1 week in vitro and then 14 d in SD conditions in soil and photographed 5 d after transfer to LD conditions. Bar = 1 cm. (**b**) C16 FA sphingolipid contents detailed according to 6 different classes (Cer, hCer, cGIPC, hGIPC, cGlCer and hGlCer). dw, Dry Weight. The means values of three biological replicates are shown ± SD. Asterisks indicate significant differences between WT control plants and indicated genotypes according to Student’s *t-*test (*P ≤ 0.05; **P ≤ 0.01; ***P ≤ 0.001).

Surprisingly, like the single *mips1* mutant, *mips1 loh2* double mutant still displayed cell death on rosette leave (Fig. 5a). In order to check sphingolipid content in this double mutant, quantification of different molecular species was performed. As expected, *loh2* single mutant displayed a strong reduction of C16 sphingolipids in all the different classes (Fig. 5b). The same reduction of C16 sphingolipids was also observed in *mips1 loh2* double mutant indicating that these sphingolipids were not involved in *mips1-*dependant PCD. The *mips1* mutant was also characterized by elevated C24 sphingolipids in particular in Cers, hCers and hGLCer. As expected from LOH2 substrate specificity, the double mutant *mips1 loh2* were still accumulating the same C24-sphingolipids (Fig. S7). Altogether, these results indicate that the HR-like *mips1* phenotype is not caused by C16 sphingolipid accumulation but is likely dependent on other sphingolipids accumulation such as C24 or could be due to another mechanism.

## Discussion

Plants are sessile organisms continuously challenged by unfavourable growth conditions caused by abiotic or biotic stress. In order to identify novel molecular regulators of this pathway, we used a genetic approach and found that the protein encoded by the *BIG* gene is required for lesion formation in the *mips1* mutant. Our work revealed that lesions triggered by a decrease in MI content could be genetically reverted in the double mutant *mips1 big* and this, in several different allelic backgrounds as *big-1*, *big-3* or *somi2*.

As demonstrated by cellular ion leakage and trypan blue staining analysis, both of which measure cell death, *somi2* is epistatic over the *mips1* mutation, indicating that BIG contributes to cell death activation in *mips1* and acts downstream of MI accumulation. The result of the quantification of phytohormones also supports this assumption. As an over-accumulation of SA in *mips1* background has been previously reported^6,15,17,35^, we have also detected an increase in JA and ABA stress-related phytohormone contents in *mips1* mutant under restrictive conditions, increase abolished by loss of function of BIG.

The fact that *somi2* was not able to suppress the root or the cotyledon developmental defects of *mips1*, confirmed our previous work indicating that these pathways can be uncoupled^15,17^. Indeed, although lesions formation was totally abolished in the *mips1 oxt6* and *mips1 hxk1* double mutants, rescue of the cotyledon phenotype was only partial and defects in root elongation were still present, suggesting that these two developmental pathways are only partly dependent on the cell death phenotype. These independent functions can be due to the role of MI as a precursor of phosphatidylinositol, a critical cellular compound involved in numerous biochemical and physiological processes, as intracellular signalling, membrane assembly and trafficking and anchoring of membrane-related proteins^1^.

How BIG can control PCD mediated by the MIPS1 pathway is difficult to decipher because the molecular function of BIG is still unknown. Indeed, the *BIG* gene encodes a 5078-aa protein with several functional domains and has been implicated in numerous hormonal and light pathways^19–21,25,27,32,36^. One hypothesis could have been that the MI content was restored in the *mips1 somi2* double mutant. This result would be consistent with our previous observations showing that a spray of MI on leaves of *mips1* mutant is enough to suppress lesion formation, indicating that inositol biosynthesis, and not the MIPS1 protein itself, is required to inhibit PCD in leaves^6^. It would also be coherent with the previous identification of *hxk1* mutant as a suppressor of *mips1*, due to a restoration of the MI content in *mips1 hxk1* mutants^15^. However, metabolomics analyses, showing that MI content were not restored in the *mips1 somi2* background, ruled out this hypothesis.

The *BIG* gene encodes a calossin-like protein possibly required for proper auxin distribution during leaf development^19,21,32^ and is specifically required in the process by which auxin inhibits endocytosis and promotes its own efflux from cells^20^. All these functions are in agreement with our results of transcriptome analysis showing an under-expression of genes involved in responses to auxin and auxin transport in *somi2* background. According to^27^, the negative consequence on auxin transport broadly observed in *big* mutants, can be brought by the JA pathway, as JA negatively restrain the turnover and the intracellular trafficking of PIN proteins and thus the auxin transport^37,38^. Although no modification of global auxin content in *somi2* background compared to WT plant has been detected, one can hypothesize that a local deregulation of auxin homeostasis or distribution can maybe suppress local cell death, as it has been recently demonstrated that DNA damage-triggered cell death in root stem cell could be largely inhibited by low levels of auxin^39^.

The demonstration of a transcriptional switch between *mips1* and its suppressor, with the reactivation of the expression of a set of genes involved in the control of cell cycle in *mips1 somi2* compared to *mips1* mutant, is likely due to a consequence of the abolition of the cell death rather than actual cause of the PCD suppression. Hence, based on these transcriptome profiling results, it is difficult to determine whether or not BIG could be a directly player of plant PCD. Indeed, although the *somi2* mutation suppresses SA, JA and ABA hyper-accumulation in *mips1*, if BIG signalling directly inhibited PCD by decreasing SA accumulation, the *somi2* mutation should have also suppressed the *mpk4* cell death phenotype that is also SA dependent^40^, a suppression that has not been observed in the double mutant *mpk4 somi2* (Fig. S8). This finding indicates that BIG’s loss of function does not antagonize all types of cell death or SA production, but rather opposes cellular responses related to plant defence mechanisms. It is consistent with the results of^36^ demonstrating that *BIG* mRNA is translationally regulated upon NB-LRR immune response activation. Using a *big* mutant challenged with the bacteria *Pst* DC3000 (AvrRpm1), authors showed that BIG is required for optimal RPM1-mediated resistance. These result were further confirmed by^27^ indicating that BIG is required for full resistance against both virulent and avirulent bacteria in Arabidopsis and that BIG deficiency promotes JA-production and restricts the accumulation of SA when challenging by these stress. It is worth noting, that we had previously demonstrated that another *mips1* suppressor, CPSF30, also contributes to *P. syringae* bacterial pathogen resistance in both basal defence and R gene mediated disease resistance pathways by positively regulating SA-Mediated Immunity in Arabidopsis^17^, indicating a point of convergence between these distinct pathways which should oppose events occurring upstream of the induction of SA production in *mips1*.

Finally, although our results gain insight into the molecular control of cell death, how MI levels regulate this process is still an open question. It has been reported that elevated levels of ceramides and of hydroxyceramides in *mips1*, correlated with the decrease of MI and of its derivative phosphatidylinositol^7^, and authors hypothesized that this could establish a pro-death signal leading to SA production. They also revealed a significant decrease of PtdIns content in *mips1* compared to wild-type plants. Specifically, PtdIns is a precursor to the inositolphophorylceramide (IPC) and GIPC synthesis and authors speculated that *mips1* mutant undergo spontaneous cell death due to a decline in PtIns availability that could limit IPC synthase activity and result in elevated ceramide and hydroxyceramide accumulation. Quantification of several molecule species of sphingolipids that we report here, confirmed an increased level of ceramide and hydroxyceramide in the *mips1* background compared to the wild-type, without any significant changes in *somi2* and *mips1 somi2* mutants, allowing a correlation between the accumulation of these sphingolipids species and the HR-like phenotype and confirming an epistatic effect of *somi2* over *mips1*. However, the increase in Cer and hCer accumulation observed in *mips1* was not detectable in *somi2 mips1* mutants, indicating that changes in ceramide accumulation are not likely a direct consequence of reduced MI availability. Hence, this result differs from the interpretation previously proposed^7^, since changes in sphingolipids homeostasis due to decreased levels of available PtIns would lead to a constitutive decrease of these contents in *mips1* mutant. Despite a specific increase of *LOH2* expression in the *mips1* background, we also reported that *mips1 loh2* double mutant still display lesions formation on leaves in the absence of C16 ceramides and hCeramides accumulation. This indicates that the C16 sphingolipid increase correlated with *LOH2* over-expression, is very likely a consequence of cell death rather than its cause and that the accumulation of C24-SL, mainly Cer, hCer and GluCer could on the other hand be responsible for *mips1* HR. However, we have observed specifically in *mips1* mutant the down-regulation of 10 genes all encoding activities of the elongase complex involved in the synthesis of VLCFAs, while one would have expected an increase in the expression of these genes regarding SL accumulation. The most likely hypothesis would be a negative feedback regulation by the accumulation of C24-SL on the VLCFAs biosynthetic pathway to maintain SL homeostasis. It is also worth noting that the two *FAH* genes are also down-regulated in *mips1* which is in agreement with the accumulation of SA that was also reported in the double *fah1 fah2* Arabidopsis mutant in correlation with an increase of ceramides contents^41^.

In conclusion, we demonstrate that, in Arabidopsis, BIG is a signalling component involved in MI-mediated cell death. In order to avoid PCD initiation during normal development and unrestricted cell death in response to infection, this process must be strictly controlled. Thanks to the intensifying finding of additional components of the SA pathway response and of their interactions among them, our knowledge of the SA-mediated defence network has been greatly improved. Moreover, the fact that cross-talks exist between SA and several other defence responses and hormone signalling pathways such as JA, has made our understanding more complex. Our understanding of the control of responses to environmental stress will be improved by the discovery of specific proteins in plant defence signalling networks. Further work will be required to reveal how the still unknown cellular function of the BIG protein can interfere with cell death signalling pathways.

## Methods

### Plants material and growth conditions

The T-DNA lines *mips1-1* (SALK_023626), *big-1* (SALK_045560), *big-3* (SALK_107817), *loh2-2* (SALK_024192), *mpk4* (SALK_056245) mutants were in the Columbia-0 (Col-0) background and *mips1-2* (Flag605F08) mutant was in the Wassilewskija (Ws) background. All these T-DNA lines were obtained from public seeds banks^42,43^. All double mutants were obtained by crossing and genotyped with appropriate primers listed in Table S3.

For primary root length and cotyledon analyses, seeds were sown on commercially available 0.5 Murashige and Skoog medium (Basalt Salt Mixture M0221; DUCHEFA) and grown 1 week in an LD growth chamber (16 h day/8 h night, 20 °C). For all other analyses, plants were grown 1 week as previously and transferred to soil under SD conditions (8 h day/16 h night, 200 μmol photons s^−1^ m^−2^, 21 °C) for 2 weeks. Plants were then subsequently transferred to LD conditions (16 h day/8 h night, 200 μmol photons s^−1^ m^-2^, 21 °C) for the times indicated.

### Bulk segregant analysis and identification of the *somi2* mutation

To map the *somi2* mutation, *mips1-1 somi2* plants in the Col-0 background, were crossed with an allelic mutant of *mips1-1*, the *mips1-2* mutant, in the WS background. First, a Bulk Segregant Analysis (BSA) was performed using F2 plants without (n = 11) or with lesions (n = 11) in LD. Primer sequences that flank polymorphism markers between Col-0 and Ws are described by^44^. Second, fine mapping of the *somi2* mutation was performed by a high throughput sequencing of nuclear DNA obtained from a bulk of 116 F2 plants without lesions in LD. Whole Arabidopsis genome re-sequencing was performed by the company MACROGEN using the ILLUMINA HiSeq2000 technology. The software CLC GENOMIC WORKBENCH (www.clcbio.com*)* was used to analyse sequences and identify single-nucleotide polymorphisms (SNP) by comparison with the Col-0 genome as reference (TAIR10). After two backcrosses of *mips1-1 somi2* with *mips1-1* single mutant to clean background mutations, Cleaved Amplified Polymorphic Sequences (CAPS) markers were used on 26 *mips1-1 somi2* plants to removed SNP mutations not linked to the *somi2* locus. Primers used are listed in Table S3.

### Cell death assays: electrolyte loss, trypan blue staining and SA quantification

Electrolytes loss, trypan blue staining and SA quantification were performed as previously described^15,17^. For electrolyte loss, leaf discs were removed with a 5 mm punch and washed in distilled water for 10 min. Six leaf discs per genotype were transferred to a tube containing 2 mL of distilled water and agitated 2 h. Conductivity was then measured with the CDM 210 conductivity meter (Radiometer Analytical) and expressed as μS per leaf surface (cm^2^). Means and SD were from four replicates per genotype per experiment.

### RNA extraction and RT-qPCR

Total RNA was extracted using the Nucleospin RNA kit (MACHEREY- NAGEL) and 2 μg of total RNA was subjected cDNA synthesis using Improm-II reverse transcriptase (A3802, PROMEGA). 1/25th of the synthesized cDNA was mixed with 100 nM solution of each primer and LightCycler 480 Sybr Green I master mix (ROCHE APPLIED SCIENCE) for quantitative PCR analysis. Products were amplified and fluorescent signals acquired with a LightCycler 480 detection system. qRT‐PCR analysis was performed as described previously^15^. Primers used are described in Table S3.

### RNA seq samples and analyses

RNA seq samples and analyses were performed mainly as described by^45^. Wild type and mutant plants were grown under SD for 3 weeks and then transferred to LD conditions during 4 days to induce lesion formation. Total RNA were obtained by pooling rosette leaves of several plants and extracted with the NucleoSpin RNA kit (MACHEREY–NAGEL), according to the manufacturer’s instructions.

RNA-seq libraries were prepared using 2 μg of total RNA with the NEBNext Poly(A) mRNA Magnetic Isolation Module and NEBNext Ultra II Directional RNA Library Prep Kit for Illumina (NEW ENGLAND BIOLABS), according to manufacturer’s recommendations.

The quality of the libraries was assessed with Agilent 2,100 Bioanalyzer (AGILENT), and the libraries were subjected to 1 X 75 bp high-throughput sequencing by NextSeq500 (ILLUMINA). Trimmomatic was used for quality trimming. The reads were mapped onto the TAIR10 assembly, using STAR. Differential analysis was done with DESeq2. Only genes for which the variations were significantly different were retained for further analysis (p value < 0.05). GO analysis were mined with the software tools AgriGO^31^.

RNAseq data from this article were deposited at Gene Expression Omnibus (https://www.ncbi.nlm.nih.gov/geo/) with accession number GSE143244.

### Metabolites and phytohormones extraction and analysis

For analytical procedures, 100 mg of fresh weight of rosette leaves of the indicated lines were harvested 4 d after transfer to LD conditions. Samples were lyophilized, ground, and metabolites were extracted. MI, scopoletin and phytohormones quantification and analysis were performed as previously described respectively by^15,17,46^. Statistical significances based on unpaired two samples Student’s t-test were determined with MICROSOFT EXCEL software.

### Sphingolipids extraction and analysis

Targeted sphingolipids analysis were performed on 3 biological repetitions on wild type and mutant plants grown under SD for 3 weeks and then transferred to LD conditions during 4 days to induce lesion formation. Sphingolipids were extracted from 2 mg freeze-dried material from rosette leaves and analyzed by UPLC-ESI–MS/MS. Extraction and chromatographic conditions, mass spectrometric parameters, and multiple reaction monitoring (MRM) methods were defined as described previously^47^. Statistical significances based on unpaired two samples Student’s t-test were determined with MICROSOFT EXCEL software.

## Supplementary information

Supplementary file1 (DOCX 16 kb)

Supplementary file2 (PDF 1180 kb)
