# Supplementary material for: Involvement of Arabidopsis BIG protein in cell death mediated by Myo-inositol homeostasis
Source: Sci Rep. 2020 Jul 9;10:11268. doi: 10.1038/s41598-020-68235-4 (PMC7347573; doi:10.1038/s41598-020-68235-4)
Supplement: Supplementary file 1 — Supplementary file1 (DOCX 16 kb) [file 41598_2020_68235_MOESM1_ESM.docx]

**Fig. S1**. Total scopoletin contents in the indicated genotypes with means and standard deviations calculated from four biological replicates grown for seven days in short days conditions and then transferred to long days for 4 days.

**Fig. S2.** Total IAA levels in the indicated genotypes, with means and SD calculated from four biological replicates grown for 14 d in SD conditions and transferred to LD conditions for 4 d (LD) or stayed in SD (SD).

**Fig. S3**. Primary root lengths of the indicated genotypes measured with means and SD calculated from 30 seedlings grown in vitro for 7 d in LD conditions

**Fig. S4.** (a) Cotyledon phenotypes of *mips1* seedlings grown under LD conditions divided into different classes according to the severity of the dimorphism. Bars = 1 mm. (b) Relative amounts of each of the cotyledon phenotypic classes in the indicated genotypes. The means values of two biological replicates are shown ± SD.

**Fig. S5**. Complementation tests between *somi2* and *big-1* and *big-3* alleles confirming that *somi2*, *big-1* and *big-3* were allelic mutations within the same gene. Plants were grown 1 week in vitro and then 14 d in SD conditions in soil and photographed 5 d after transfer to LD conditions.

**Fig. S6: Principle component analysis (PCA) of the RNA-seq samples.** PCA reveals the samples of the same genotype clustered together, whereas the samples of different genotypes were separated based on the distance between each sample along the first two principal components. Each dot represents one sample, and the dots with the same colour represent the samples of the same genotype.

**Fig. S7.** C24 FA sphingolipids contents in *mips1*, *loh2* and *mips1 loh2* double mutants compared to the wild-type Col-0 detailed according to 6 different classes (Cer, hCer, cGIPC, hGIPC, cGlCer and hGlCer). DW, Dry Weight. The means values of three biological replicates are shown ± SD. Asterisks indicate significant differences between WT control plants and indicated genotypes according to Student’s *t-*test (*, *P* ≤ 0.05; **, *P* ≤ 0.01; ***, *P*≤ 0.001).

**Fig. S8**. *somi2*-mediated suppression of cell death is not able to rescue the phenotype of *mpk4* mutant. Rosette leaf phenotypes of the indicated genotypes. Plants were grown one week *in vitro*, then 14 days in SD conditions in soil and photographed seven days after transfer to LD conditions. Scale bars represent 1cm in each panel. Details of Trypan blue staining of wild-type, *mips1*, *mpk4,* and *mips1 mpk4* leaves are shown in the thumbnail picture. Bars = 2 mm.
