# Supplementary material for: Involvement of Arabidopsis BIG protein in cell death mediated by Myo-inositol homeostasis
Source: Sci Rep. 2020 Jul 9;10:11268. doi: 10.1038/s41598-020-68235-4 (PMC7347573; doi:10.1038/s41598-020-68235-4)
Supplement: Supplementary file 2 — Supplementary file2 (PDF 1180 kb) [file 41598_2020_68235_MOESM2_ESM.pdf]

SUPPLEMENTARIES INFORMATIONS

Involvement of Arabidopsis BIG protein in cell death mediated by *Myo*-Inositol homeostasis

Quentin Bruggeman[1,2], Florence Piron-Prunier[1,2], Frédérique Tellier[3], Jean-Denis Faure[3], David Latrasse[1,2], Deborah Manza-Mianza[1,2], Christelle Mazubert[1,2], Sylvie Citerne[3], Stéphanie Boutet-Mercey[3], Raphael Lugan[4], Catherine Bergounioux[1,2], Cécile Raynaud[1,2], Moussa Benhamed[1,2] and Marianne Delarue[1,2] *.

Fig. S1

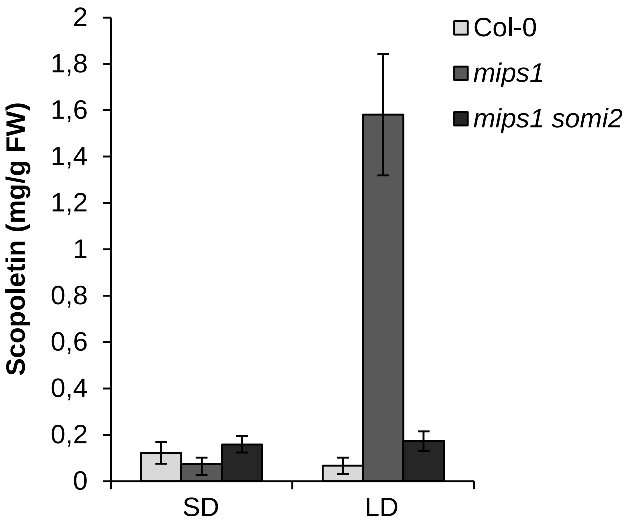

Fig. S2

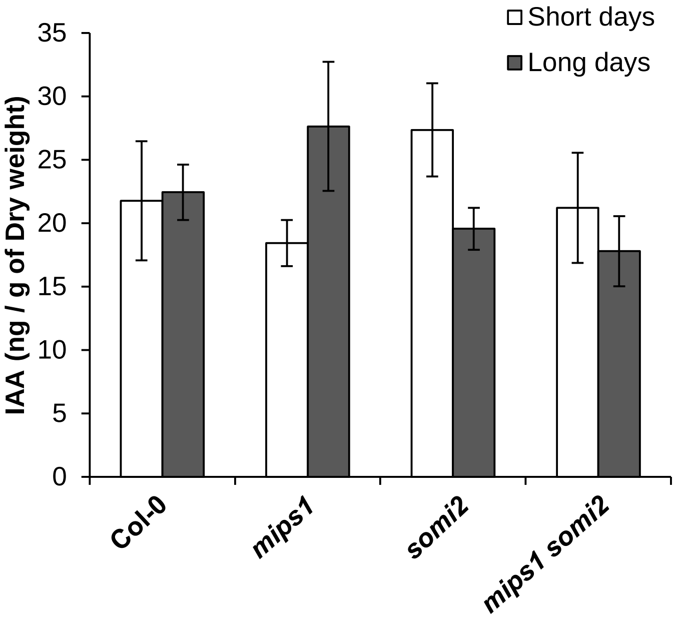

Fig. S3

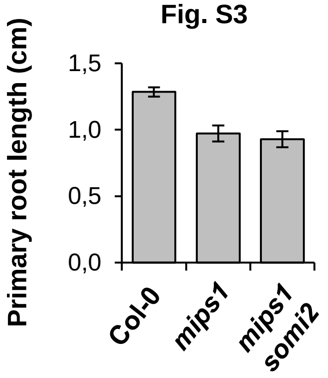

Fig. S4

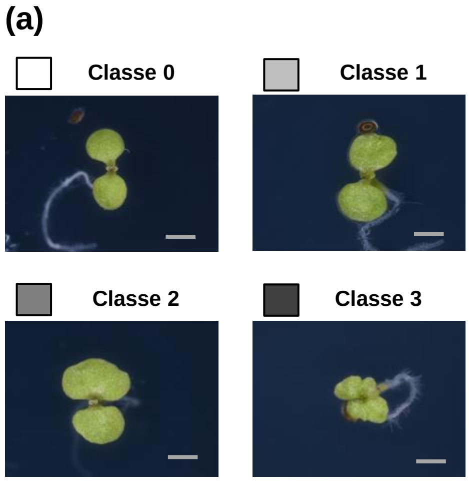

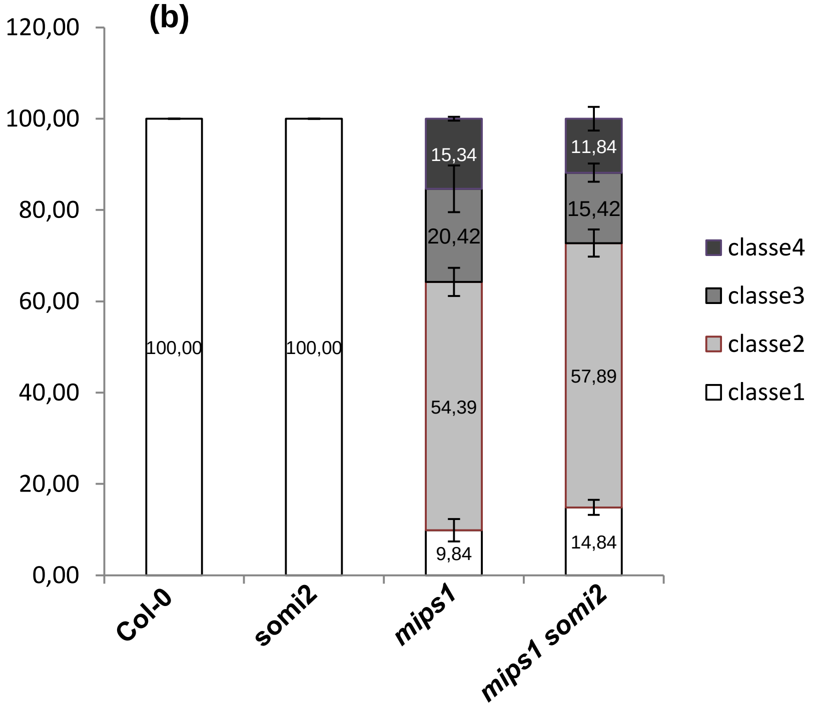

Fig. S5

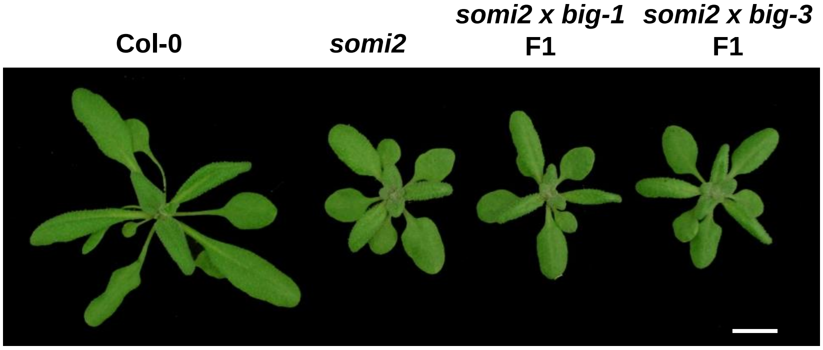

Fig. S6

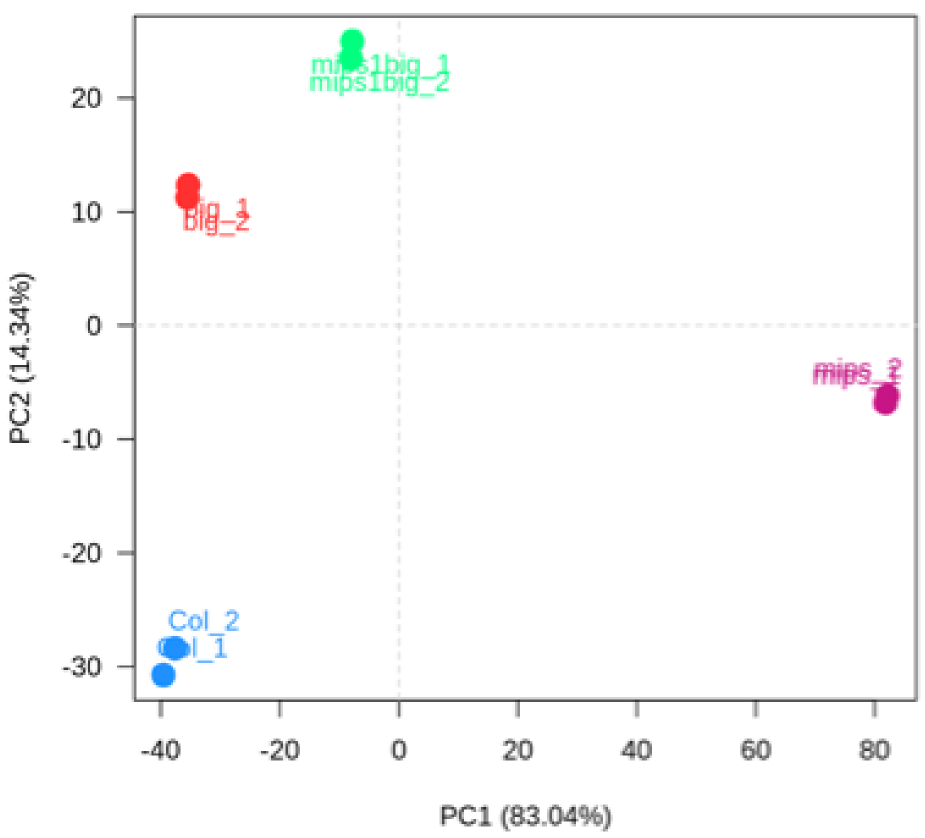
mips1big_1
mips1big_2
big_1
big_2
mips_2
mips_1
Col_2
Col_1
PC2 (14.34%)
PC1 (83.04%)
20
10
0
-10
-20
-30
-40
-20
0
20
40
60
80

Fig. S7

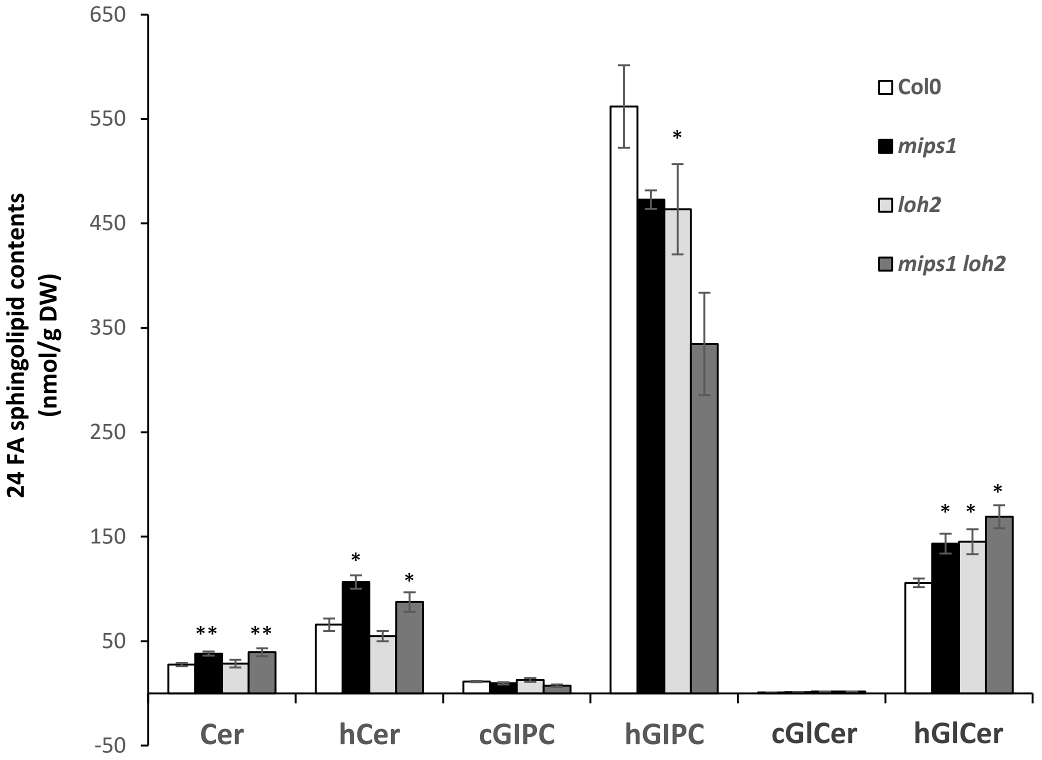

650
550
450
350
250
150
50
-50
24 FA sphingolipid contents (nmol/g DW)
Col0
mips1
loh2
mips1 loh2
**
**
*
*
*
*
*
*
Cer
hCer
cGIPC
hGIPC
cGlCer
hGlCer

Fig. S8

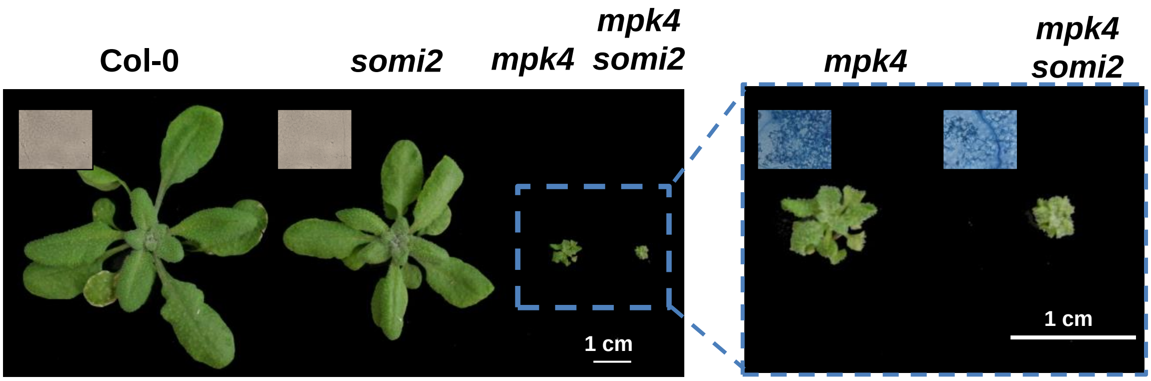

TABLE S1

List of genes specifically under-expressed in *mips1* involved in sphingolipids metabolism

Genes	Description
AT1G01120	KCS1 (3-ketoacyl-CoA synthase 1)
AT2G26250	KCS10 (3-ketoacyl-CoA synthase 10)
AT2G26640	KCS11 (3-ketoacyl-CoA synthase 11)
AT2G46720	KCS13 (3-ketoacyl-CoA synthase 13)
AT5G04530	KCS19 (3-ketoacyl-CoA synthase 19)
AT1G04220	KCS2 (3-ketoacyl-CoA synthase 2)
AT1G25450	KCS5 (3-ketoacyl-CoA synthase 5)
AT3G55360	ECERIFERUM 10 (CER10), Enoyl-CoA reductase
AT2G34770	FAH1 (Fatty acid hydroxylase 1)
AT4G20870	FAH2 (Fatty acid hydroxylase 2)
AT5G10480	PASTICCINO 2 (PAS2) (Protein tyrosine phosphatase-like)
AT1G14290	SPHINGOID BASE HYDROXYLASE 2 (SBH2)

TABLE S2

List of genes under-expressed in *mips1* and over-expressed in *somi2* and *mips1 somi2* enriched for GO corresponding to "Cell Cycle" terms

Genes	Description
AT5G65420	CYCLIN D4;1
AT5G03270	CYCLIN D6
AT2G45080	CYCLIN p3
AT2G38620	CYCLIN dependent kinase B1 ;2
AT3G50410	OBF Binding protein 1
AT1G76310	CYCLIN B2;4
AT1G07370	PCNA
AT2G42260	UV-B Insensitive 4
AT2G33560	BUB1-related (BUB1: budding uninhibited by benzymidazol 1)
AT4G34160	CYCLIN D3;1
AT4G35620	Cyclin B2;2
AT4G21270	kinesin 1
AT2G17620	Cyclin B2;1
AT3G23670	phragmoplast-associated kinesin-related protein
AT1G16330	cyclin b3;1
AT1G20610	Cyclin B2;3
AT3G57860	UV-B-insensitive 4-like protein
AT3G25980	DNA-binding HORMA family protein
AT1G44110	Cyclin A1;1
AT1G02065	squamosa promoter binding protein-like 8

AT3G11520	CYCLIN B1;3
AT1G03780	targeting protein for XKLP2
AT1G15570	CYCLIN A2;3
AT4G05190	kinesin 5
AT2G26760	Cyclin B1;4
AT5G51600	Microtubule associated protein (MAP65/ASE1) family protein
AT5G06150	Cyclin family protein
AT1G76540	cyclin-dependent kinase B2;1

TABLE S3

RT-qPCR primers

Primer name	DNA sequence (5' to 3')
PR1-fwd	ACTACAACTACGCTGCGAACAC
PR1-rev	TCACTTTGGCACATCCGAGTC
PR5-fwd	TGTCGTGGCCTGCAAGAGTG
PR5-rev	AGTCCGTGGGAGGACAAGTTTC
BIG-fwd	CCGTAGGGTCTTTCTCCAGC
BIG-rev	CGGTTAAGGTTTCTGCGTCTTG
UBQ10-fwd	GGCCTTGTATAATCCCTGATGAATAAG
UBQ10-rev	AAAGAGATAACAGGAACGGAAACATAGT

Genotyping primers

Primer name	DNA sequence (5' to 3')
Lba1	TGGTTCACGTAGTGGGCCATCG
Tag3	TGATACCAGACGTTGCCCGCATAA
MIPS1-LP	TTGCTAGCAACCATATCGTCC
MIPS1-RP	TTCGTGTCGGATCTTTTAACG
MIPS1-2-RP	ACTCACACCCCAAAACCGATCCA
BIG-LP1	ACTGGTGCTGATGAGATCCAC
BIG-RP1	AAAGTGGAGAGGACGTATTCCAG
BIG-LP3	ATTTGTATGACAAACAGCCGCTC
BIG-RP3	CTTTATTGAGAGCCCCAGTCC
MPK4-RP	GTGACAATGCAAGAAGATACGTTAGACAGC
MPK4-LP	CTTGAAATATCTACAGAGTTGGTGTG
LOH2-RP	AAACCGCACTGGCACATATAG
LOH2-LP	TCCACTGTTATCCTATTATCTTCGC

Couple of primers for genotyping mutant lines

T-DNA line	WT allele	Mutant allele
mips1-1	MIPS1-LP + MIPS1-RP	MIPS1-RP+Lba1
mips1-2	MIPS1-LP + MIPS1-2-RP	MIPS1-RP + Tag3
big-1	BIG-LP1 + BIG-RP1	BIG-RP1 + Lba1
big-3	BIG-LP3 + BIG-RP3	BIG-RP3 + Lba1
loh2-2	LOH2-LP + LOH2-RP	LOH2-RP + Lba1
mpk4	MPK4-LP + MPK4-RP	MPK4-RP + Lba1

CAPS primers

Primer name	DNA sequence (5' to 3')
XPO1b-caps-fwd	AATGACTGTATATCTTCTTTACCTTTAAG
XPO1b-caps-rev	CAAATGCCAACCATCATCCCTCG
AT3G05870-caps-fwd	GCAGAAGAGAATGGCAGTTCAAAGAGTAAC
AT3G05870-caps-rev	AGCAATCAAACCAAGGACAAGC
AT3G07980-caps-fwd	CAACGATCCGAGATACTTGAC
AT3G07980-caps-rev	TTTGATGCTTCTTGGTTGTTAATTTCGTCG

Couples of primers for CAPS identification

Gene	CAPS PCR	Digestion enzyme
XPO1	XPO1b-caps-fwd + XPO1b-caps-rev	HindIII
AT3G05870	AT3G05870-caps-fwd + AT3G05870-caps-rev	Dde1
AT3G07980	AT3G07980-caps-fwd + AT3G07980-caps-rev	Sal1
